# Adipose-Derived Mesenchymal Stem Cells Restore Impaired Mucosal Immune Responses in Aged Mice

**DOI:** 10.1371/journal.pone.0148185

**Published:** 2016-02-03

**Authors:** Kazuyoshi Aso, Akitoshi Tsuruhara, Kentaro Takagaki, Katsuyuki Oki, Megumi Ota, Yasuhiro Nose, Hideki Tanemura, Naoki Urushihata, Jinichi Sasanuma, Masayuki Sano, Atsuyuki Hirano, Rio Aso, Jerry R. McGhee, Kohtaro Fujihashi

**Affiliations:** 1 Department of Pediatric Dentistry, The University of Alabama at Birmingham, Birmingham, AL, United States of America; 2 BioMimetics Sympathies Inc., Tokyo, Japan; 3 Department of Neurosurgery, Shinyurigaoka General Hospital, Kawasaki, Japan; 4 Sunfield Clinic, Koto-ku, Tokyo, Japan; The Ohio State University, UNITED STATES

## Abstract

It has been shown that adipose-derived mesenchymal stem cells (AMSCs) can differentiate into adipocytes, chondrocytes and osteoblasts. Several clinical trials have shown the ability of AMSCs to regenerate these differentiated cell types. Age-associated dysregulation of the gastrointestinal (GI) immune system has been well documented. Our previous studies showed that impaired mucosal immunity in the GI tract occurs earlier during agingthan is seen in the systemic compartment. In this study, we examined the potential of AMSCs to restore the GI mucosal immune system in aged mice. Aged (>18 mo old) mice were adoptively transferred with AMSCs. Two weeks later, mice were orally immunized with ovalbumin (OVA) plus cholera toxin (CT) three times at weekly intervals. Seven days after the final immunization, when fecal extract samples and plasma were subjected to OVA- and CT-B-specific ELISA, elevated levels of mucosal secretory IgA (SIgA) and plasma IgG antibody (Ab) responses were noted in aged mouse recipients. Similar results were also seen aged mice which received AMSCs at one year of age. When cytokine production was examined, OVA-stimulated Peyer’s patch CD4^+^ T cells produced increased levels of IL-4. Further, CD4^+^ T cells from the lamina propria revealed elevated levels of IL-4 and IFN-γ production. In contrast, aged mice without AMSC transfer showed essentially no OVA- or CT-B-specific mucosal SIgA or plasma IgG Ab or cytokine responses. Of importance, fecal extracts from AMSC transferred aged mice showed neutralization activity to CT intoxication. These results suggest that AMSCs can restore impaired mucosal immunity in the GI tract of aged mice.

## Introduction

Immune functions deteriorate with age in several species [1–6]. In humans, the elderly are at a higher risk for infections, especially influenza virus and *Streptococcus pneumoniae*, as well as for certain autoimmune diseases and cancer, and their immune responses to vaccination are diminished [7–11]. It is well established that aged humans exhibit loss of naïve T cells and exhibit a more restricted T cell repertoire [6]. Further, aging results in decreased human CD8^+^ cytotoxic T lymphocyte (CTL) responses, restricted B cell clonal diversity, failure to produce high affinity antibodies (Abs), and an increase in memory T (T_M_) cells. It has been suggested that although certain dendritic cell (DC) populations are fully functional in aging, both foreign- and self-antigens (Ags) induce enhanced proinflammatory cytokines [2, 5, 10, 12]. This enhanced inflammation can be detrimental; however, very old individuals with a more balanced pro- and anti- inflammatory phenotype may be the most fortunate. The association of inflammation in aging has been termed “inflamm aging”; however, we still do not have direct evidence that inflamm aging occurs in and therefore influences the mucosal immune system [13].

Studies have provided extensive evidence of dysregulation and of an overall decline in mucosal immunity, especially in the gastrointestinal (GI) tract of the elderly [14, 15]. The most common method for evaluating mucosal immune responses is perhaps to test external secretions for the presence of secretory IgA (SIgA) antibodies (Abs). In humans, GI tract lavages taken from either aged or young subjects were shown to contain similar total Ig levels. Our group has shown that early development of aging occurs in the GI tract immune system of mice [14]. Thus, fecal extract samples from one-year old mice contained low levels of Ag-specific SIgA Abs; however, total IgA levels were essentially the same as those seen in young adult mice [14]. Further, reduced numbers of naïve CD4^+^ T cells and follicular DCs (FDCs) were associated with diminished sizes of Peyer’s patches of one-year old mice [16].

Adipose-derived mesenchymal stem cells (AMSCs) are attractive since they can be readily obtained and expanded. It has been shown that AMSCs can differentiate into adipocytes, chondrocytes, and osteoblasts [17]. In addition, various clinical trials have shown the regenerative capacity of AMSCs [18–23]. Previous studies showed a therapeutic potential for AMSCs for Alzheimer’s [24, 25] and periodontal disease [24, 25]. In this study, we hypothesized that AMSCs could restore an impaired mucosal immune system in aged mice. Thus, we have employed an oral immunization strategy to examine Ag-specific mucosal immune responses in aged mice adoptively transferred with AMSCs.

## Materials and Methods

### Mice

C57BL/6 mice were purchased from the Frederick Cancer Research Facility (National Cancer Institute, Frederick, MD). Upon receipt, mice were transferred to microisolators and maintained in horizontal laminar flow cabinets and provided sterile food and water *ad libitum*. Experiments were performed using young adult mice between 6 to 8 weeks of age or aged mice either 12–14 months old (~ one year), or mice over 18 months of age. All experiments involving mice were performed in accordance with both NIH and the University of Alabama at Birmingham (UAB) Institutional Animal Care and Use Committee (IACUC) guidelines. UAB IACUC gave specific approval for all procedures involving mice; animal protocol number 09853.

### Adoptive transfer of AMSCs

AMSCs were isolated from adipose tissue of C57BL/6 mice (mAMSCs) or a female human subject (hAMSCs) by a combination of enzymatic digestion and centrifugation. All procedures involving human subjects were approved by the Sun Field Clinic Institutional Review Board (IRB). Adult human adipose tissues were collected from volunteers undergoing orthopedic surgery following the ethical guidelines of the Sun Field Clinic in Tokyo, JAPAN. Informed consent was obtained from the volunteers before the surgical procedure. The isolated stromal vascular fraction was passed through 70 μm filter and cultured in serum-free medium (BioMimetics Sympathies Inc., Tokyo JAPAN) for expansion. A total of 2 x 10^6^ AMSCs were adoptively transferred into 12 or 18 months old mice intraperitoneally. Two weeks or 8–10 months later, mice were orally immunized with OVA plus cholera toxin (CT) as described below.

### Oral immunization

Aged mice with or without AMSC transfer and young adult mice were immunized three times at weekly intervals with oral doses of 1 mg of OVA (Fraction V; Sigma, St. Louis, MO) and 10 μg of CT (List Biological Laboratories, Campbell, CA) in PBS [14, 26].

Animals were monitored on a daily basis until humane endpoint. If alterations in physical presentation or behavorial deficiencies occur, the mice were humanely euthanized. None of the mice in this study died prior to humane endpoint. Plasma and fecal extracts were collected one week after the last oral immunization. Further, mice were sacrificed at the same point using CO_2_ followed by the cervical dislocation. Mononuclear cells from spleen and intestinal lamina propria (LP) were isolated, and subjected to OVA-specific ELISPOT assays [14, 26]. Further, CD4^+^ T cells isolated from spleen, LP and Peyer’s patches (PPs) were examined for antigen (Ag)-specific cytokine responses.

### Antibody assays

Antibody (Ab) titers in plasma and fecal extracts were determined by an enzyme-linked immunosorbent assay (ELISA). Falcon Microtest assay plates (Becton Dickinson, Oxnard, CA) were coated with an optimal concentration of OVA (100 μl of 1 mg /ml) or recombinant CT-B (100 μl of 5 μg /ml of CT-B: List Biological Laboratories) in PBS [14, 26]. In order to detect Ag-specific Ab levels, horseradish peroxidase- conjugated, goat anti-mouse μ, γ or α heavy chain-specific Abs [Southern Biotechnology Associates, (SBA), Birmingham, AL] were employed. Endpoint titers were expressed as the last dilution yielding an optical density at 414 nm (OD_414_) of > 0.1 units above negative control values after a 15 min incubation period.

### Enumeration of antibody-forming cells

The spleen was removed aseptically and single-cell suspensions prepared as described elsewhere [14, 26]. After being carefully excised from the intestinal wall, PPs were dissociated using collagenase type V (0.5 mg/ml, Sigma) to obtain single-cell preparations. Mononuclear cells in the LP were isolated after removal of PPs from the small intestine using a combination of enzymatic dissociation and discontinuous Percoll gradients (Pharmacia Fine Chemicals, Uppsala, Sweden) [14, 26]. Mononuclear cells in the interface between the 40% and 75% layers were removed, washed and resuspended in RPMI 1640 (Cellgro Mediatech, Washington, DC) supplemented with HEPES buffer (15 mM), L-glutamine (2 mM), penicillin (100 U/ml), and streptomycin (100 μg/ml) and 10% FCS (complete medium). An ELISPOT assay was employed to detect cells producing IgM, IgG and IgA Abs. Ninety-six-well nitrocellulose plates (Millititer HA, Millipore Corp., Bedford, MA) were coated with goat anti-mouse Ig [SBA] at 2 μg / ml (100 μl / well) to detect total IgM, IgG and IgA Ab-forming cells (AFC), 1 mg / ml of OVA for anti-OVA specific AFCs, or 5 μg / ml of CT-B (100 μl /well) for anti-CT-B specific AFCs.

### Antigen-specific CD4^+^ T cell responses

CD4^+^ T cells were purified by the magnetic activated cell sorter system (Miltenyl Biotec Inc., Sunnyvale, CA) as described previously [27, 28]. Briefly, cells were incubated in a nylon wool column (Polysciences Inc., Warrington, PA) to remove B cells and macrophages. Enriched T cell populations were then incubated with biotinylated anti-CD4 (GK 1.5) mAb followed by streptavidin-conjugated microbeads and passed through a magnetized column. The purified T cell fractions were > 97% CD4^+^ and were > 99% viable. Cells were resuspended in complete medium and purified CD4^+^ T cells (4 x 106 cells/ml) were cultured with or without 1 mg/ml of OVA in the presence of T cell-depleted, irradiated (3000 Rads) splenic Ag-presenting cells (APCs). These APCs were derived from naïve mice and were placed in 96-well or 24-well tissue culture plates (Corning Glass Works, Corning, NY) for 5 days at 37°C in a moist atmosphere of 5% CO2 in air. In some experiments, culture supernatants were harvested after 2 or 5 days of incubation and were then subjected to a cytokine-specific ELISA.

### Cytokine-specific ELISA

Levels of cytokines in culture supernatants were measured by ELISA. The details of the ELISA for IFN-γ and IL-4 have been described previously [28, 29]. For coating and detection, the following mAbs were used: for anti-IFN-γ: R4-6A2 and XMG 1.2 mAbs; for anti-IL-4: BVD4-1D11 and BVD6-24G2 mAbs. The levels of Ag-specific cytokine production were calculated by subtracting the results of control cultures (e.g., without Ag stimulation) from those of Ag-stimulated cultures. This ELISA was capable of detecting 0.78 ng / ml of IFN-γ; 23.4 pg / ml of IL-4. For intracellular cytokine analysis, cells were incubated with ionomycin (1 μg/ml; Sigma-Aldrich) and phorbol 12-myristate 13-acetate (PMA, 25 ng/ml; Sigma-Aldrich) for 3 hr in the presence of Monensin and then stained with FITC-conjugated anti-CD4 mAb (BD Biosciences), before being stained intracellularly with PE-labeled anti- IFN-γ or IL-4 mAbs (BD Biosciences). The samples were then subjected to FACS analysis (FACS Calibur; BD Biosciences).

### Functional properties of AMSC restored SIgA Abs

To examine if Ag-specific SIgA Abs induced in aged mice with AMSC transfer possess actual neutralization activity, the mouse jejunum loop test was performed [30]. The jejunum of naïve young adult mice were ligated with a piece of cotton thread at a distance of about 4 to 6 cm from the pylorus. Immediately after ligation, each loop was injected with 200 μl of fecal extract samples and 20 μl of CT (1 mg/ml) or PBS. After 12 hours, each loop was hung on a fixed clip and stretched by placing another clip on the other end of the loop. Then, the length and weight of each loop were measured. The volume/length ratio (μl/cm) was used to express the intensity of the reaction.

### Statistics

The significance of the difference (e.g., *p* values) between groups was evaluated by the Mann Whitney U test using a Statview II program designed for Macintosh computers.

## Results

### Restoration of Ag-specific Ab responses in aged mice given AMSCs

**Fig 1:**
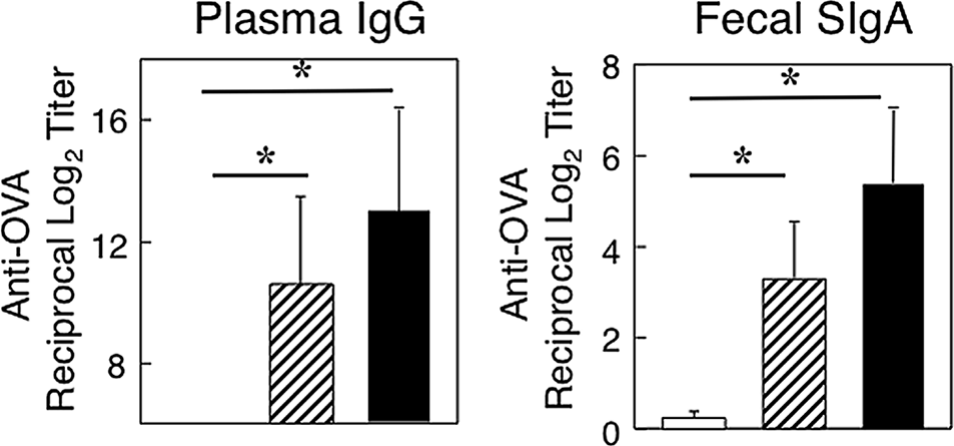
OVA-specific Ab responses in aged and young adult mice. Aged mice were adoptively transferred with mAMSCs as described in the Materials And Methods section. Aged mice given mAMSCs (hatched bar), naïve aged mice (without mAMSC transfer, open bar) and young adult mice (closed bar) were orally immunized with 1 mg OVA plus 10 μg of CT three times at weekly intervals. One week after the last immunization, levels of anti-OVA fecal SIgA and plasma IgG Abs were determined by OVA-specific ELISA. The values shown are the mean ± SEM taken from 10 mice in each group. *p < 0.05 when compared with aged mice without AMSC adoptive transfer.

**Fig 2:**
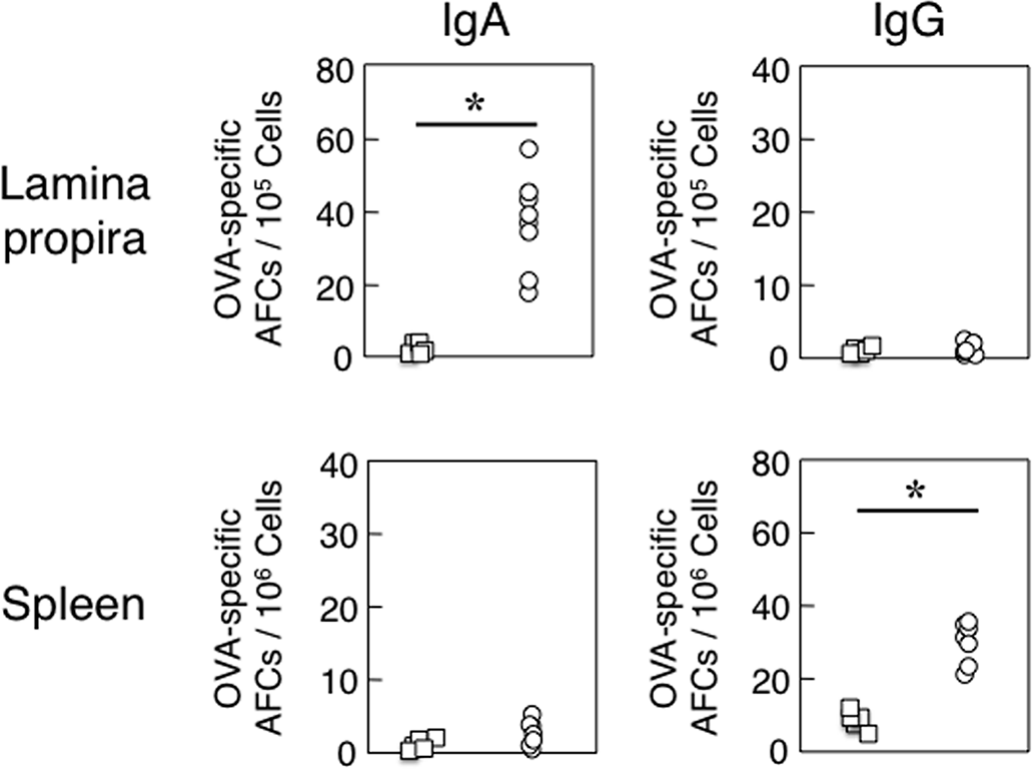
OVA-specific AFCs in lamina propria (LP) and spleen of aged mice. Aged mice with (○) or without (□) mAMSC transfer were orally immunized as described in Fig 1 legend. One week after the last immunization, mononuclear cells were isolated from the LP and spleen and were then subjected to an OVA-specific ELISPOT assay to determine the numbers of IgA and IgG AFCs. Naïve mice served as a control group and did not exhibit any anti-OVA AFCs (data not shown). The values shown are the mean ± SEM taken from 10 mice in each group. *p < 0.05 when compared with aged mice without adoptive transfer of mAMSCs.

We initially examined OVA-specific immune responses in aged mice with or without mAMSC transfer. Aged mice following mAMSC transfer showed increased levels of OVA-specific plasma IgG Ab responses when compared with those responses seen in mice without transfer (Fig 1). Further, elevated levels of OVA-specific SIgA Ab responses were noted in fecal extracts of aged mice with the mAMSC transfer when compared with those in orally immunized aged mice without adoptive transfer (Fig 1). Interestingly, the levels of OVA-specific SIgA Ab responses in aged mice with mAMSC transfer were similar to those seen in young adult mice orally immunized with OVA plus CT (Fig 1). In order to confirm these OVA-specific Ab response results, we next determined the numbers of AFCs in lamina propria and spleen by OVA-specific ELISPOT assay. Increased numbers of OVA-specific IgA AFCs were seen in the LP of aged mice with mAMSC transfer (Fig 2). Further, increased numbers of OVA-specific IgG AFCs were noted in spleen of aged mice after mAMSC transfer (Fig 2). These results clearly show that OVA-specific Ab responses are restored in aged mice after adoptive transfer with mAMSCs.

**Fig 3:**
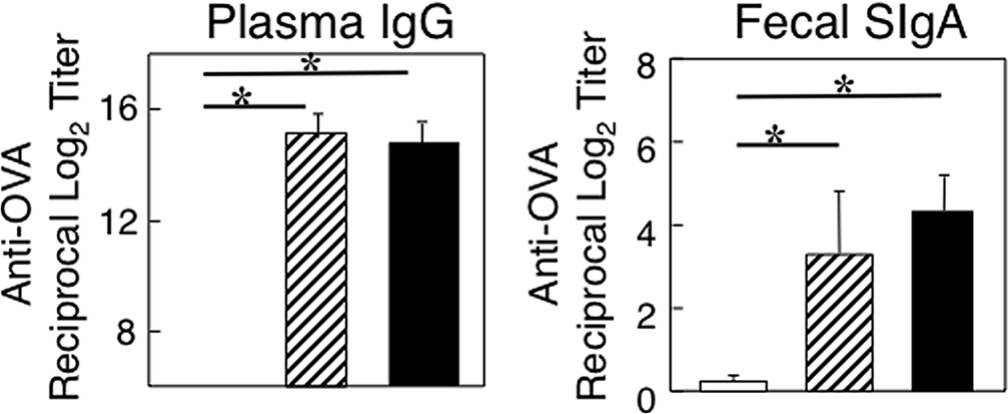
OVA-specific Ab responses in aged mice given human AMSCs (hAMSCs). Aged mice were adoptively transferred with hAMSCs. Aged mice adoptively transferred with hAMSCs (hatched bar), naïve aged mice (without hAMSC transfer, open bar) and young adult mice (closed bar) were orally immunized with 1 mg of OVA plus 10 μg CT three times at weekly intervals. One week after the last immunization, levels of anti-OVA fecal SIgA and plasma IgG Abs were determined by an OVA-specific ELISA. The values shown are the mean ± SEM taken from 10 mice in each group. *p < 0.05 when compared with aged mice without AMSC adoptive transfer.

In some experiments, hAMSCs were adoptively transferred into two-year old mice and the mice were orally immunized with OVA plus CT as mucosal adjuvant. Aged mice given hAMSCs showed increased levels of anti-OVA mucosal SIgA and plasma IgG Ab responses when compared with orally immunized aged mice without hAMSC adoptive transfer. These responses were essentially the same as those seen in young adult mice given oral OVA plus CT (Fig 3). Of importance, although hAMSCs expressed MHC class I molecules, they did not exhibit any MHC class II expression (S1 Fig). In this regard, we have never observed any allogenic reactions when hAMSCs were adoptively transferred into recipient mice. Further, when hAMSCs were co-cultured with mouse splenic cells, no proliferative responses were induced (S2 Fig). These findings are consistent with previous reports by other groups, which showed that human umbilical cord stem cells and adipose tissue-derived stem cells do not express MHC class II or co-stimulatory molecules. Further, these stem cells moderately expressed MHC class I, but were poorly immunogenic as they failed to lead to any significant allogenic reaction [31, 32]. In addition, others showed that *in vitro* expanded, adipose-derived mesenchymal cells retained low immunogenicity [33].

### CT-B specific Ab responses occur in aged mice with AMSCs

**Fig 4:**
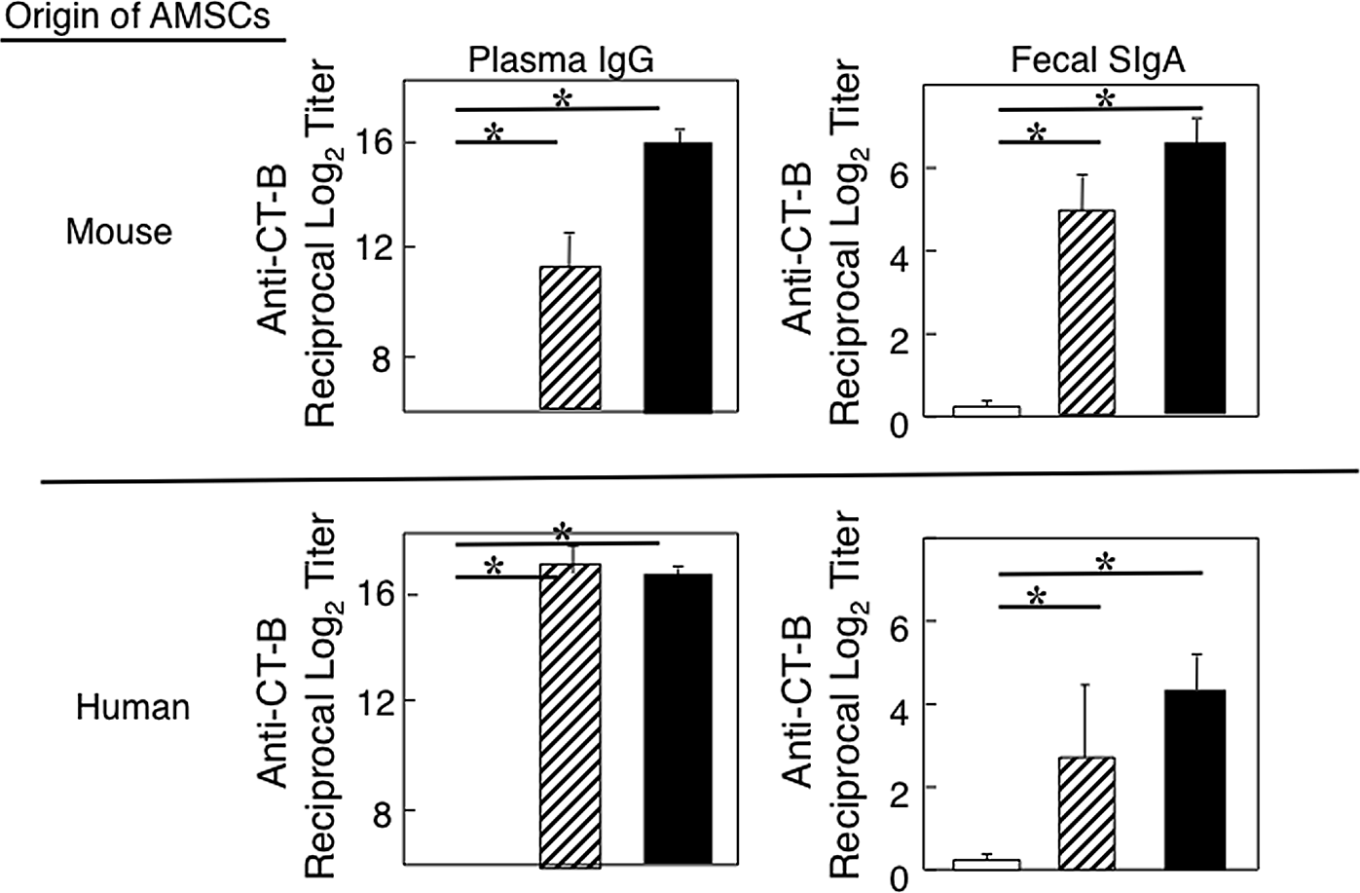
CT-B-specific Ab responses in aged and young adult mice. Aged mice were adoptively transferred with mAMSCs or hAMSCs. Aged mice given mAMSCs (hatched bar), naïve aged mice (without hAMSC transfer, open bar) and young adult mice (closed bar) were orally immunized with 1 mg of OVA plus 10 μg of CT three times at weekly intervals. One week after the last immunization, levels of anti-CT-B fecal SIgA and plasma IgG Abs were determined by CT-B-specific ELISA. The values shown are the mean ± SEM taken from 10 mice in each group. *p < 0.05 when compared with aged mice without either mAMSC or hAMSC transfer.

It was important to test whether immune responses to CT were also restored in aged mice with AMSC transfer since CT is a potent mucosal Ag as well as an adjuvant. In this study, mAMSC or hAMSC transferred aged mice given oral OVA and CT 3 times at weekly intervals showed significantly high levels of CT-B-specific IgA and IgG Ab responses in fecal extracts and plasma samples, respectively (Fig 4). These responses were comparable to those seen in young adult mice given oral OVA plus CT. On the other hand, these mucosal and systemic anti-CT-B Ab responses were reduced in aged mice without either mAMSC or hAMSC transfer. These results show that even strong Ag such as CT fails to elicit specific Ab responses in the mucosal and systemic compartments of aged mice; however, AMSC transfer can restore impaired CT-B-specific immune responses.

### Assessment of intracellular CD4^+^ Th1- and Th2-type cytokine responses

**Fig 5:**
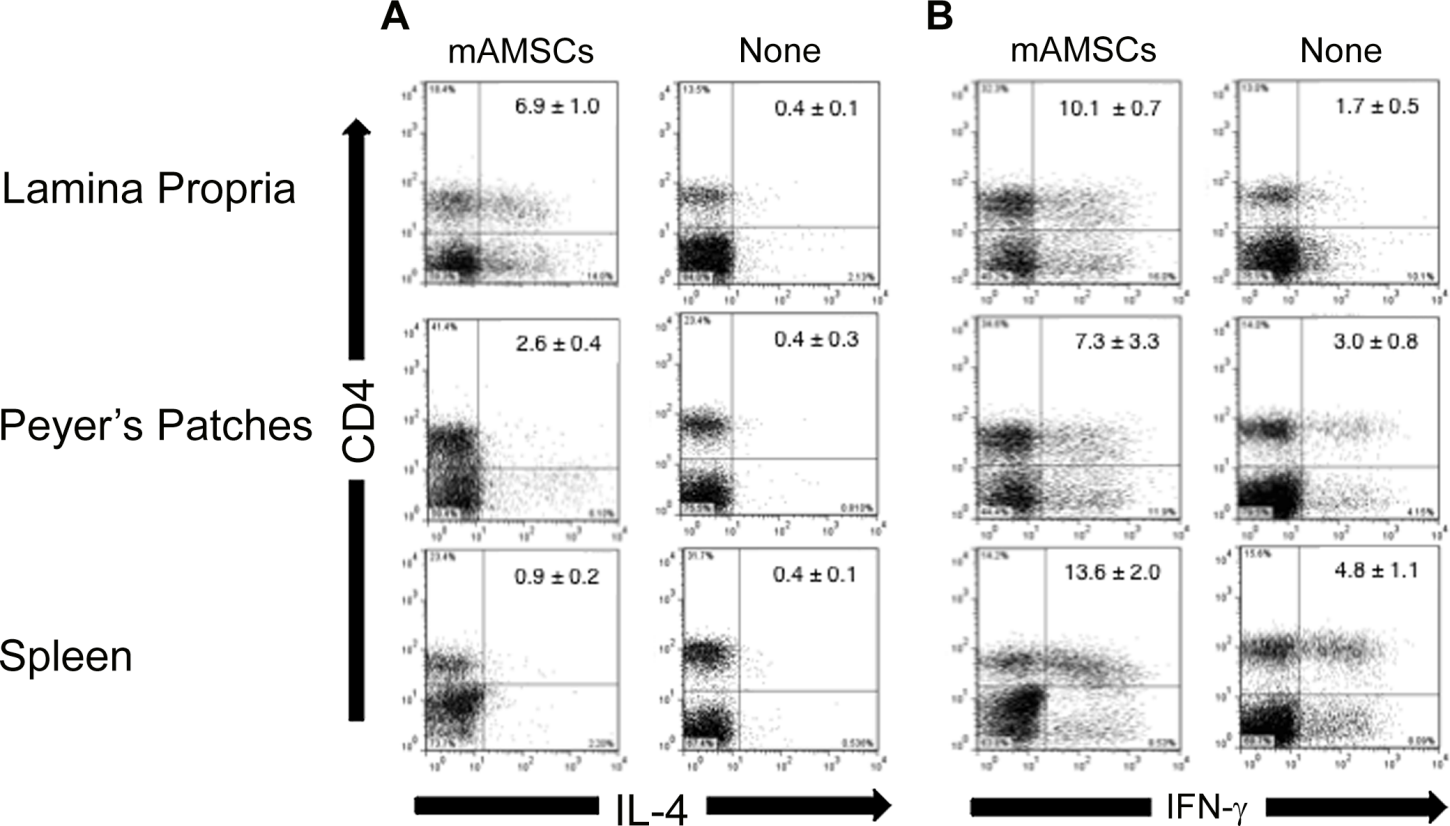
Interleukin-4 and IFN-γ production by CD4^+^ T cells from PPs, LP and spleen were determined by intracellular analysis. Mononuclear cells were isolated from aged mice given oral OVA plus CT with or without mAMSC adoptive transfer. Cells were incubated with ionomycin (1 μg/ml) and phorbol 12-myristate 13-acetate (PMA; 25 ng/ml) for 3 hr and then stained with FITC-labeled anti-CD4 mAb. Samples were further stained intracellularly with PE-labeled anti-IL-4 (A) or anti-IFN-γ (B) mAb. The frequencies of cytokine-producing cells were determined by flow cytometry. The profiles represent typical results and are taken from one of three separate experiments.

We next determined levels of Th1- and Th2-type cytokine responses by CD4^+^ T cells. Mononuclear cells from LP, PPs and spleen were stimulated with PMA and ionomycin for 4 hr. Cells were then stained with anti-CD4 mAb followed by intracellular IFN-γ or IL-4 staining. Increased frequencies of IL-4 producing CD4^+^ T cells were seen in LP, PPs and spleen of mice given mAMSCs when compared with those from mice without AMSC transfer. Among these tissues, the LP contained the highest numbers of IL-4 producing CD4^+^ T cells (Fig 5A). In contrast, the spleen showed minimal numbers of IL-4 producing CD4^+^ T cells. When the numbers of IFN-γ-producing CD4^+^ T cells were examined, LP, PPs and spleen of mAMSC adoptively transferred mice contained significantly higher frequencies of IFN-γ-producing CD4^+^ T cells than those in mice without mAMSC transfer (Fig 5B). These results show that impaired CD4^+^ Th1- and Th2-type cytokine responses in aged mice were restored after mAMSC transfer.

### Ag-stimulated Th1- and Th2-type cytokine responses by CD4^+^ T cells

**Fig 6:**
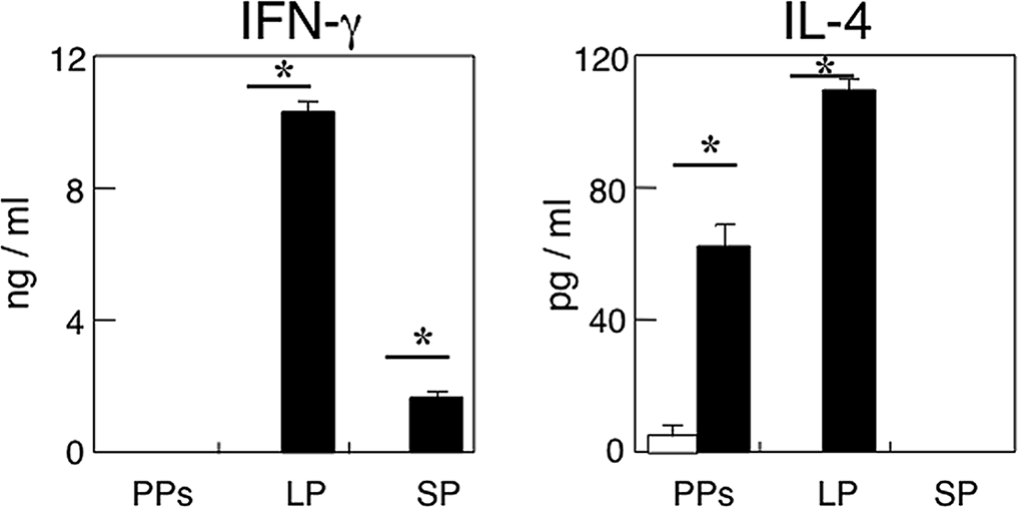
Th1- and Th2-type cytokine production by CD4^+^ T cells. The CD4^+^ T cells (4 x 10^6^ cells/ml) from aged mice given oral OVA plus CT with (closed bars) or without (open bars) mAMSC transfer were purified from PPs, LP, and spleen one week after the final immunization. Cells were then cultured with 1 mg/ml OVA in the presence of irradiated splenic antigen-presenting cells (8 x 10^6^ cells/ml). Culture supernatants were harvested after 5 days of incubation and analyzed by the respective cytokine-specific ELISA. The levels of Ag-stimulated cytokine production were calculated by subtraction of the results of culture without OVA stimulation from those with OVA stimulation. The values shown are the mean ± SEM of 10 mice in each experimental group. *p < 0.05 when compared with aged mice without AMSC transfer.

Since our previous studies showed that IL-4 production by Ag-specific CD4^+^ T cells play an essential role in the induction of SIgA Ab responses when CT was employed as a mucosal adjuvant [34, 35], we next examined Ag-stimulated Th1- and Th2-type cytokine responses in aged mice with or without mAMSC transfer. Purified CD4^+^ T cells from LP, PPs and spleen were cultured with or without OVA in the presence of Ag-presenting cells for 5 days. The culture supernatants were harvested and subjected to IL-4- and IFN-γ- specific ELISAs. The levels of Ag-specific cytokine production were calculated by subtracting the results of culture without OVA-stimulation from those with OVA stimulated cultures. OVA-stimulated PP CD4^+^ T cells from mAMSC transferred mice revealed increased levels of IL-4 production when compared with those from mice without adoptive transfer (Fig 6). Further, CD4^+^ T cells from LP showed elevated levels of IL-4 and IFN-γ production (Fig 6). These results clearly indicate that IL-4 -producing CD4^+^ T cells in mucosal inductive and effector tissues play key roles in the induction of Ag-specific SIgA Ab responses.

### Functional properties of SIgA Abs

**Fig 7:**
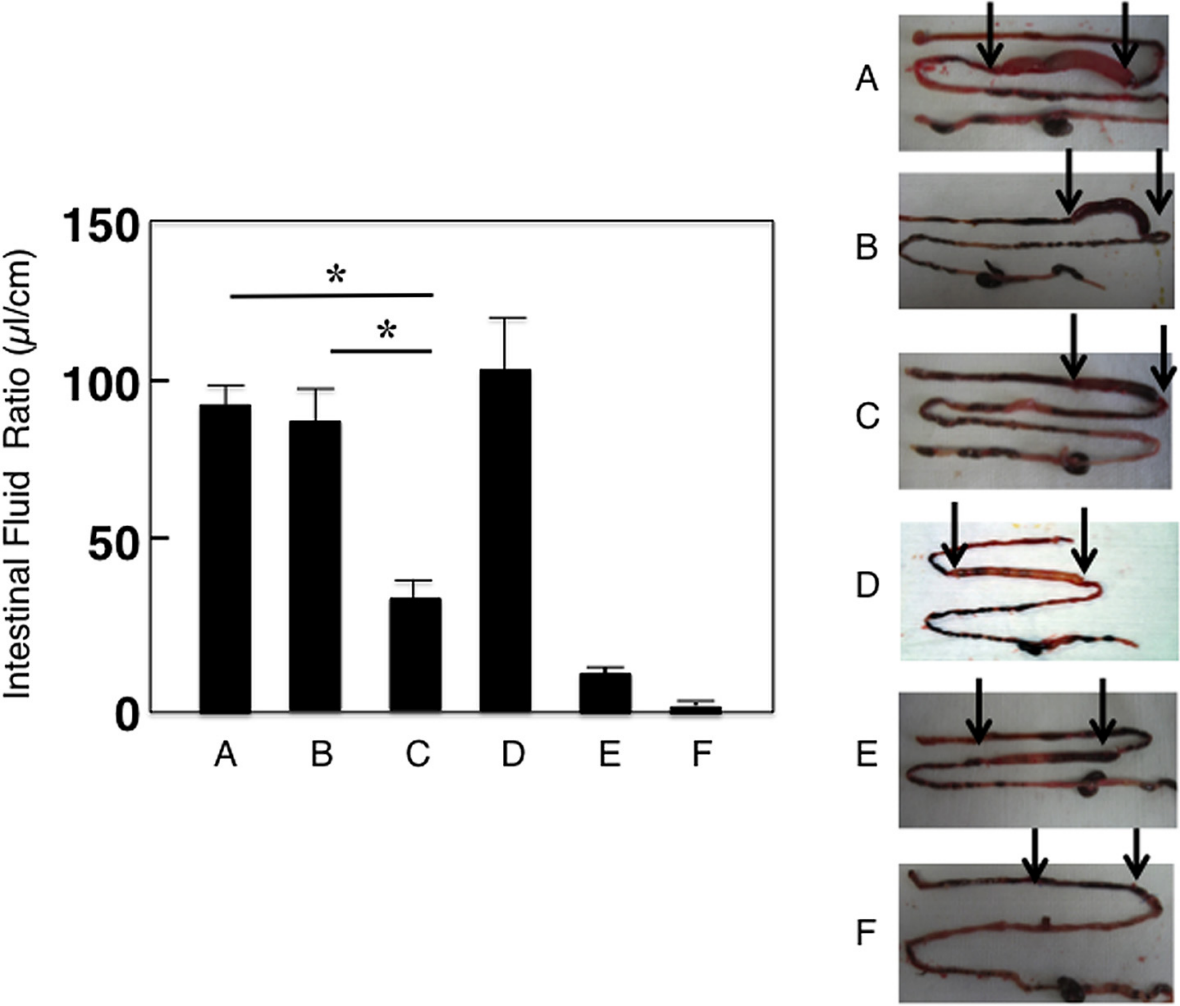
Functional properties of CT-B-specific SIgA Abs in aged mice adoptively transferred with AMSCs. Each fecal extract sample (200 μl) from (A and F) naïve aged mice, (B) aged mice given oral OVA plus CT, (C) AMSC adoptively transferred aged mice given oral OVA plus CT, (D) naïve young adult mice or (E) young adult mice given oral OVA plus CT, was injected into the ligation of jejunum loops (4–6 cm) of mice together with 20 μl of CT (1 mg/ml]) (A-E) or (F) PBS. Twelve hours after injection, each loop was hung on a fixed clip and stretched by placing another clip on the other end of the loop. The length and weight of each loop were measured. The volume/length ratio (μl / cm) was used to express the intensity of the reaction. The results represent the mean values ± SEM of 6 mice in each experimental group. *p < 0.05 when compared with aged mice without mAMSC adoptive transfer or naïve aged mice. The pictures represent typical results and are taken from one of three separate experiments.

It is important to assess whether SIgA Abs in aged mice restored by AMSC adoptive transfer could provide protective immunity to CT intoxication. Fecal extracts from AMSCs transferred aged mice revealed significant neutralization activity to CT intoxication. Thus, markedly reduced levels of intestinal fluid accumulation were noted when compared with those levels induced by injection of CT mixed with fecal extracts from naïve aged or from young adult mice (Fig 7). In contrast, jejunum loop injection of CT mixed with fecal extracts from immunized, aged mice without AMSC transfer showed essentially the same levels of fluid efflux as those seen in negative controls. These results suggest that CT-B-specific SIgA Abs in aged mice restored by AMSC transfer exhibit functional activity for neutralization of CT intoxication.

## Discussion

The present study is the first to show that AMSCs restore impaired mucosal and systemic immune responses in both partially and completely aged mice. This is a unique and new feature of AMSCs which contrasts with previous studies generally showing that MSCs down regulate various immunocompetent cells [36–38]. For example, MSCs inhibited both CD4^+^ and CD8^+^ T cell proliferation following co-culture and polyclonal stimulation [36–38]. Other *in vitro* studies showed reduced Abs in mixed lymphocyte cultures [36–38] as well as reduced B cell proliferation and Ab synthesis [36–38]. Finally, co-culture of MSCs with splenic B cells induced regulatory B cells producing IL-10 that ameliorated autoimmunity and Ab synthesis [36–38]. Our new system has taken a separate approach which allows AMSCs to restore an impaired mucosal immune system. Thus, Ag-specific SIgA Ab responses were significantly increased in aged mice adoptively transferred with AMSCs when orally immunized with OVA and CT. The induction of Ag-specific SIgA Ab responses was supported by increased levels of IL-4 production in mucosal tissues of aged mice which was achieved by pre-treatment with AMSCs. Of importance, Ag-specific SIgA Abs in aged mice restored by AMSC transfer were functional. Thus, fecal extracts containing CT-B-specific SIgA Abs exhibited neutralization activity against CT intoxication. In summary, the major difference with studies of others is that we assessed AMSC functions by adoptive transfer *in vivo* in a mouse model. Further, we employed serum-free medium to expand AMSCs. Thus, transferred AMSCs and their soluble products may totally differ from others and upregulate various immune competent cells.

One of the features of immunosenescence is an increased threshold of inflammation known as “inflamm aging” [13]. Thus, chronic inflammatory responses may hamper induction of Ag-specific immune responses when active immunization was initiated, since it was essential to induce a transient inflammatory innate immune responses in order to elicit subsequent acquired immunity [39]. It has been shown that MSCs also exhibited potential roles for anti-inflammatory functions [40]. Thus, MSCs have been employed as therapeutic strategies for various immune disorders including graft-versus-host disease (GVHD) [41–43], organ transplantation [44–47], autoimmune diseases [48–52] and inflammatory bowel disease [53, 54]. Indeed, MSCs interact with T cells to reduce their pro-inflammatory cytokine production [36, 55], while increasing their production of anti-inflammatory cytokines, including IL-4 and IL-10 [56, 57]. In this regard, we considered that AMSC transfer into aged mice could reduce inflamm aging and facilitate the subsequent restoration of Ag-specific immune responses when mice were orally immunized with OVA and CT. Of importance, our present study noted that increased numbers of IL-4 producing CD4^+^ T cells with increased levels of OVA-induced IL-4 production by CD4^+^ T cells in PPs. Since IL-4 is an essential Th2-type cytokine for adjuvant activity of CT [34, 35], these results clearly indicate that AMSCs enhanced IL-4 production in aged mice, which could also potentially down-regulate inflammatory responses and simultaneously allow CT to enhance OVA-specific Ab responses.

We also noted increased levels of IFN-γ production in aged mice following AMSC transfer when orally immunized with OVA plus CT. Our previous studies showed that although CT as mucosal adjuvant elicited dominant co-administered Ag-specific Th2-type cytokine responses in the mucosal inductive tissues including PPs and NALT, increased levels of IFN-γ production was induced due to the part of its own adjuvant function [16, 58]. Indeed, our current study showed that OVA-induced IL-4 but not IFN-γ production was detected in PPs of aged mice with AMSC transfer despite increased numbers of IFN-γ producing CD4^+^ T cells in aged mice with AMSC transfer. In contrast, CD4^+^ T cells in the LP produced significant levels of both IFN-γ and IL-4 after *in vitro* OVA stimulation, which agrees with our previous findings [59]. Taken together, these results support the notion that AMSC transfer restores impaired immune responses in aged mice to normal levels as those seen in young adult mice. Indeed, aged mice without AMSC transfer failed to induce significant levels of either IL-4 and IFN-γ production by CD4^+^ T cells when orally immunized with OVA plus CT.

In addition to interacting with T and B cells, MSCs also play regulatory roles for innate immune cells including monocytes, macrophages and dendritic cells (DCs) [60–65]. Notably, DCs co-cultured with MSCs showed aberrant maturation, cytokine production and down-regulation of activated T cells [60–62, 64]. Of interest, when mature DCs which expressed high levels of CD11c, MHC class II and co-stimulatory molecules were co-cultured with MSCs, these DCs lost these surface molecules, and exhibited CD11b molecule expression and phagocytic activity, which characterize immature DCs [64]. Although *in vitro* MSC-treated mature DCs failed to induce T cell proliferation, this DC population expressed CD11c and MHC class II when adoptively transferred into recipient mice [64]. These results suggest that *in vivo* interactions of MSCs and mature DCs may induce an immature phenotype of DCs with Ag-presenting cell (APC) function for the activation of naïve T cells and subsequent Ag-specific Ab responses. Even though *in vitro* MSC-treated, mature DCs express Jagged-2 which is essential for down-regulation of activated T cells [64], it has been shown that Jagged-2 expressed by DCs interacts with Notch expressing naïve CD4^+^ and thus plays a central role for the induction of Th2-type effector CD4^+^ T cells. Indeed, our current findings showed that adoptive transfer of AMSCs prior to oral immunization elicited IL-4 producing, Ag-specific CD4^+^ T cells in aged mice. Thus, it is possible that AMSCs interact with mature DCs to regenerate immature DCs for increased total numbers of APCs in order to restore impaired mucosal immune responses in aged mice. To support this view, our previous studies showed that a DC targeting mucosal adjuvant consisting of a plasmid encoding Flt3 ligand and CpG ODN successfully induced Ag-specific SIgA Ab responses in aged mice [27, 66, 67]. Studies to address these issues are currently under investigation.

In the present study, we showed that AMSC transfer is a potent immune therapy for restoration of mucosal immunity. Thus, AMSCs which may potentially down-regulate pre-existing inflammatory responses by enhancing APC functions regulate the induction of DC-mediated mucosal immunity. However, other cellular and molecular mechanisms by AMSCs are also likely required for the restoration of immunosenescence. It has been suggested that major changes associated with aging are the decline in the gut immune system and the changes in the distribution of the intestinal microbiota [15]. In this regard, it is possible that AMSC adoptive transfer re-shapes the intestinal microflora of aged mice to more resemble to that seen in young adult mice. To test this hypothesis, we are currently characterizing the microbiota of aged mice provided AMSCs via adoptive transfer. In addition to the various immunomodulatory functions for MSCs, our present findings provide a novel anti-aging ability of AMSCs to restore functions in the GI tract mucosal immune system. It is essential to further study the precise cellular and molecular mechanisms where AMSCs restore the mucosal immune system in aging in order to expand this knowledge to potential clinical applications.

## Supporting Information

S1 Fig MHC class I and class II expression by hAMSCs. hAMSCs and human peripheral blood mononuclear cells (hPBMCs) were stained with PE-conjugated either anti-human MHC class I (HLA-A, B, C; W6/32) or anti-human MHC class II (HLA-DR; L243) mAb and were then subjected to flow cytometry analysis. Black lines showed cells without staining as negative controls.
(TIFF)

S2 Fig hAMSCs failed to induce an *in vitro* allogenic reaction. hAMSCs (5 x 10^4^ /ml or 2.5 x 10^5^/ml) were cultured with mouse splenocytes (1 x 10^6^ /ml) for 2 days. During the last 24 hr of incubation, EdU (10 μg/ml) was added to each well. As negative controls, mouse splenocytes were incubated in the absence of hAMSCs. The wells containing mouse splenocytes stimulated with Con A (10 μg/ml) and PHA-P (20 μg/ml) for 2 days served as positive controls. Cells were harvested and were then stained with PerCP/Cy5.5-labeled anti-mouse CD3 mAb followed by incubation with Alexa Fluor^®^ 488-azide using an EdU Click-IT^®^ assay kit. These samples were subjected to flow cytometry analysis in order to detect incorporated EdU.
(TIFF)
