# Supplementary figures and images for: Adipose-Derived Mesenchymal Stem Cells Restore Impaired Mucosal Immune Responses in Aged Mice
Source: PLoS One. 2016 Feb 3;11(2):e0148185. doi: 10.1371/journal.pone.0148185 (PMC4740412; doi:10.1371/journal.pone.0148185)

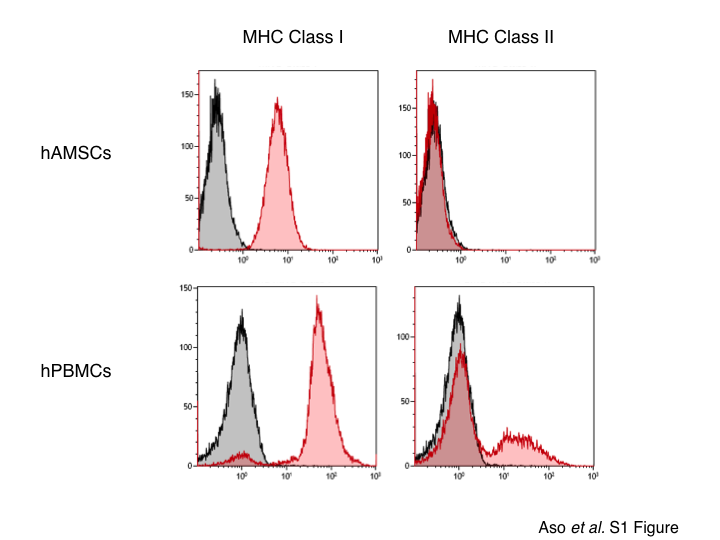

Supplement: S1 Fig — hAMSCs and human peripheral blood mononuclear cells (hPBMCs) were stained with PE-conjugated either anti-human MHC class I (HLA-A, B, C; W6/32) or anti-human MHC class II (HLA-DR; L243) mAb and were then subjected to flow cytometry analysis. Black lines showed cells without staining as negative controls. (TIFF) [file pone.0148185.s001.tiff]

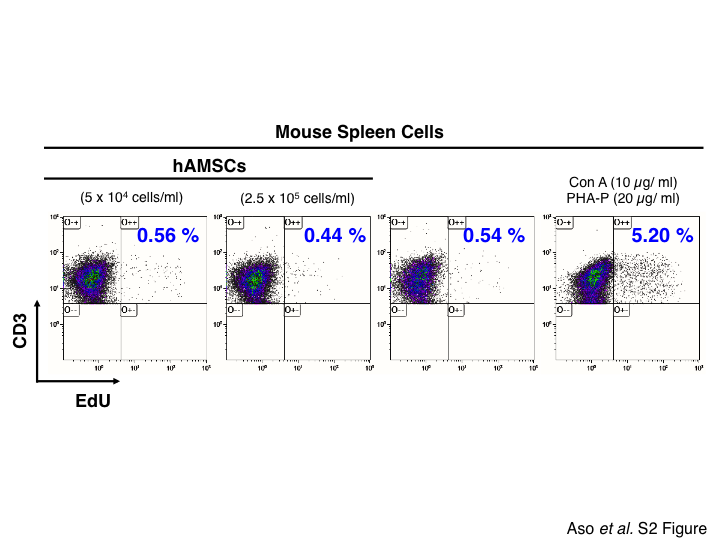

Supplement: S2 Fig — hAMSCs (5 x 10^4^ /ml or 2.5 x 10^5^/ml) were cultured with mouse splenocytes (1 x 10^6^ /ml) for 2 days. During the last 24 hr of incubation, EdU (10 μg/ml) was added to each well. As negative controls, mouse splenocytes were incubated in the absence of hAMSCs. The wells containing mouse splenocytes stimulated with Con A (10 μg/ml) and PHA-P (20 μg/ml) for 2 days served as positive controls. Cells were harvested and were then stained with PerCP/Cy5.5-labeled anti-mouse CD3 mAb followed by incubation with Alexa Fluor® 488-azide using an EdU Click-IT® assay kit. These samples were subjected to flow cytometry analysis in order to detect incorporated EdU. (TIFF) [file pone.0148185.s002.tiff]
